# Lung Point-of-Care Ultrasound for Unexpected Hypoxemia during Anesthesia

**DOI:** 10.3390/healthcare9121727

**Published:** 2021-12-13

**Authors:** Jui-Ting Wang, I-Min Su, Hsiang-Ning Luk, Phil B. Tsai

**Affiliations:** 1 Department of Anesthesia, Hualien Tzu-Chi Medical Center, Hualien 97002, Taiwan; iamp5tray@gmail.com (J.-T.W.); 100311034@gms.tcu.edu.tw (I.-M.S.); 2 Department of Anesthesiology, Rancho Los Amigos National Rehabilitation Center, Downey, CA 90242, USA; ptsai@dhs.lacounty.gov

**Keywords:** POCUS, pneumonia, hypoxemia, anesthesia

## Abstract

This is a case report showing acute hypoxemia during anesthesia. Immediate differentiation using lung POCUS (point-of-care ultrasound), in addition to physical examination and portable chest radiography, was made. This is the first case report of sputum impaction due to pneumonia causing hypoxemia that has been detected by lung POCUS during anesthesia.

## 1. Introduction

Lung point-of-care ultrasound (POCUS) facilitates the timely, convenient, and accurate bedside diagnosis of emergent respiratory conditions, such as pneumothorax, pleural effusion, and pneumonia [1]. During the COVID-19 pandemic, lung POCUS has also played a role in screening for and the diagnosis of COVID-19 pneumonia, especially in hospital settings such as the emergency room, isolation intensive care unit, and operating theatre. At the same time, unexpected acute arterial desaturation and hypoxemia during anesthesia is not uncommon [2] and often needs quick differentiation from all possibilities, such as malposition or malfunction of the endotracheal tube, mechanical problems of ventilators and breathing circuits, and patients’ cardiopulmonary dysfunctions (e.g., tension pneumothorax, secretion impaction, pleural effusion, and tamponade) [3]. In this report, we would like to demonstrate the role of lung POCUS in differentiating unexpected severe hypoxemia and bronchial mucus impaction (due to pneumonia) from tension pneumothorax in an intubated patient in the operating room.

## 2. Case Report

A 74-year-old man, ASA physical class III (163 cm, 73 kg, BMI 27.4), was scheduled for tumor-wide excision, mandibulotomy, tracheostomy, and free flap reconstruction because of mouth floor squamous cell carcinoma. His medical history included hypertension and previous cystolitholapaxy for bilateral ureteral stones. The patient was taking losartan and hydrochlorothiazide and denied any drug allergies. A pre-operative chest radiograph (10 days before the surgery) showed a normal picture and an echocardiogram indicated normal left ventricular function. A mild productive cough was noted.

A standard monitoring set-up (electrocardiogram, blood pressure, and SpO_2_) was implemented before induction of anesthesia. Pre-operative vital signs were within normal range (heart rate, 74 bpm; blood pressure, 168/85 mmHg; respiration rate, 18 times per minute; and an oxygen saturation of 94% on room air). Following pre-oxygenation, general anesthesia was induced with remifentanil (target-controlled infusion: 3 ng/mL), lidocaine (20 mg), propofol (180 mg), and succinylcholine (80 mg). Oral tracheal intubation with a 7.5 mm endotracheal tube was performed using a video-assisted intubating stylet (Trachway^®^, Markstein Sichtec Medical Corp, Taichung, Taiwan). Airway secretion was found during the tracheal intubation procedure. Mechanical ventilation was set at a volume-controlled mode with the following settings: tidal volume (500 mL), respiratory rate (10 times per minute), and positive end-expiratory pressure (PEEP; 4 cmH_2_O). Sevoflurane at an end-expiration concentration of 2% and cis-atracurium were used for the maintenance of anesthesia. An arterial line was established through a radial artery for continuous beat-to-beat monitoring.

A cuffed 8.0 sized tracheostomy tube (Rota-Trach^TM^, Vitaltec, Taichung, Taiwan, ID 8.0 mm, OD 11.0 mm, TL 76 mm) was placed after an uneventful tracheostomy procedure. The endotracheal tube was then withdrawn without incident and no secretion impaction inside the tube was noted. However, SpO_2_ dropped from 100% to 96% during the course. An immediate recruitment maneuver with FiO_2_ 1.0 was performed. When the tracheostomy was completed and ready for the second stage of the neck dissection procedure, the patient’s SpO_2_ continued to drop down from 96% to 89%. Peak airway pressure increased from 22 to 29 cmH_2_O, and EtCO_2_ decreased from 42 mmHg to 37 mmHg. Blood pressure was within normal range. Pure oxygen was given, and arterial blood gas (ABG) was checked, which revealed a low PaO_2_ (140 mmHg under FiO_2_ 1.0).

Meanwhile, physical examinations revealed that chest wall movement was normal at the left side but significantly suppressed at the right side. Lung auscultation revealed significantly diminished breath sounds over the right chest with a mixture of coarse crackles and rhonchi. Malposition of the tracheostomy tube with one-lung ventilation was immediately excluded. Since the pre-operative chest radiograph taken 10 days before was normal and the patient did not present any infection signs (fever, leukocytosis, etc.), pneumothorax was an initial impression ready to be excluded. A bedside lung POCUS with a curved transducer (2.5 to 7.5 MHz) was immediately used to evaluate the lung images.

**Figure 1:**
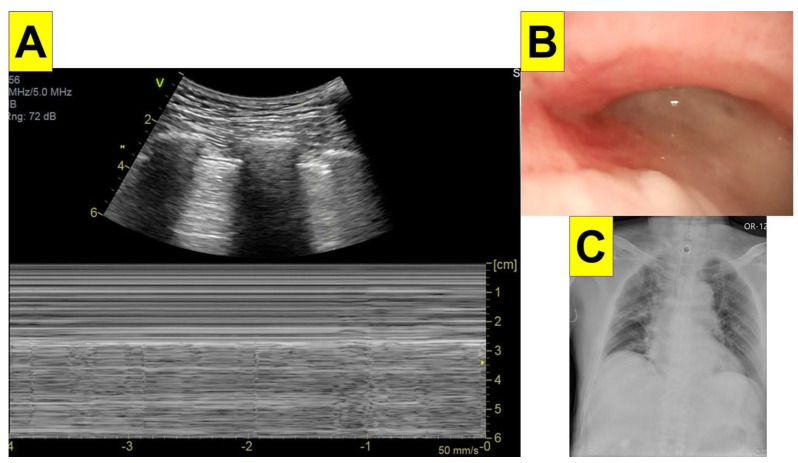
Clinical findings of acute hypoxemia during anesthesia. (**A**) Lung POCUS image; (**B**) bronchoscopic findings via tracheostomy tube; (**C**) portable chest radiograph taken shortly after the incident.

The images of the lung POCUS showed decreasing lung sliding over the right upper lung field and a reduced sandy seashore sign with intermittent lung pulse on motion mode (M-mode; Figure 1A). B-line was not observed within the 2nd and 5th intercostal space. Right mainstem intubation by the tracheostomy tube was excluded by fiberoptic bronchoscopy exam (the tip was within the trachea and above the carina). While advancing the bronchoscope past the right main bronchus, however, copious sticky yellowish sputum was encountered in the bronchial branches, especially at the right upper bronchus (Figure 1B). A portable chest radiography was taken and showed partial atelectasis over the right upper lobe and increased interstitial lung density, which were compatible with the ultrasound findings (Figure 1C). After adequate suctioning and recruitment maneuvers, an ABG analysis was repeated (PaO_2_ 115 mmHg under FiO_2_ 0.5). Because of suspected pneumonia and the planned long duration of the operation, the surgery was then deferred. Sputum culture was collected and empirical antimicrobial treatment with piperacillin and tazobactam was initiated. Haemophilus *influenza* was isolated from the sputum culture. After a 10-day antibiotic treatment course, a repeat chest radiograph was clear, and the patient received the pre-planned surgery uneventfully.

## 3. Discussion

In this report, we presented a case with a sudden onset of severe hypoxemia during an early phase of anesthesia. Lung POCUS was applied to promptly differentiate possible causes, e.g., pneumothorax and atelectasis, and facilitated critical clinical decision making.

Pre-operative pneumonia significantly increased postoperative morbidity and mortality rates [4]. Elective surgery should be delayed until pneumonia is fully resolved. However, in this case, the pre-operative chest radiograph revealed a normal picture and there were no outstanding clinical symptoms, signs, or laboratory findings (except a productive cough). Community-acquired pneumonia (CAP) caused by Haemophilus *influenza* is common and chest radiography is a critical element in diagnosing pneumonia. However, early in the disease course, chest radiography may be negative.

In our case report, copious sputum accumulation resulted in the obstructive atelectasis, which eventually led to desaturation and hypoxemia. Although abnormal findings were immediately detected (such as decreasing SpO_2_ and EtCO_2_, increasing peak airway pressure, depressed chest wall movement, and abnormal auscultation findings), other causes (e.g., tension pneumothorax) needed to be quickly excluded before a portable chest radiograph could be taken in time.

Since decades ago, POCUS (including lung ultrasonograph) has emerged as a valuable bedside diagnostic tool to facilitate clinical diagnosis in a timely manner, provide guidance, and direct appropriate rapid therapeutic management [5,6]. Lung POCUS modality has been commonly used in emergency departments and intensive care units when a regular follow-up of lung conditions is needed. It is a noninvasive and irradiative diagnostic tool and can be performed by experienced and trained medical specialists other than radiologists (e.g., pulmonologists, intensivists, emergency doctors, and anesthesiologists) [7,8]. The sensitivity and specificity of POCUS to diagnose acute critical conditions, such as pneumothorax, pneumonia, acute respiratory distress syndrome, pulmonary edema, or embolism, have been compared with those of other gold-standard diagnostic modalities recently.

Although the absence of lung sliding (visceral pleural sliding on the parietal pleura, showing as a glistening movement at the pleural line) suggested pneumothorax, an identified lung point (a sharp demarcation between abolished and normal lung slide following respiration and a boundary of aerated lung tissues and collected air space) may support the diagnosis of pneumothorax with high specificity. Meanwhile, dynamic imaging recording depicting lung pulse (the synchronized motion of the pleura with the heart beating cycle transmitted through lung tissue) and B-lines (or comet-tail artifact) may rule out pneumothorax. Instead of showing a typical seashore sign, pneumothorax presents typical barcode sign (or stratosphere sign; parallel horizontal lines above and below the pleural line) under the M-mode. Another contrasting scenario to be differentiated is pneumonia. While supporting clinical findings are integrated, prompt diagnosis of pneumonia could be made if lung POCUS showed lung consolidation, patchy B-lines (or lung rocket sign; associated with atelectasis or pulmonary edema), or dynamic air-bronchograms. In the presence of marked consolidation, echo features and signs such as the loss of a clear pleural line, tissue-like patter with air-bronchograms, shredded sign (fractal sign; an irregular serrated border between the aerated lung and the consolidated lung), or hyperechogenic areas could be observed.

In this case, we performed a bilateral anterior chest echography via the 2nd–5th intercostal space at the bilateral mid-axillary line. Several sonographic signs and features were recognized. Abolished lung sliding was found by curved transducer (2.5 to 7.5 MHz) and a reduced seashore sign was further recorded by M-Mode (Figure 1A). A reduced seashore sign with abolished lung sliding, combined with a decreased breathing sound, points to decreased lung movements. The absence of a B-line (or lung rocket sign) means no significant congestion or consolidation. While lung pulse was observed, a lung point sign (high specificity for pneumothorax) was not seen in this case. As described in the decision tree by the BLUE protocol, the A’ profile should be followed [6]. Since a lung point was not observed, other diagnostic modalities (e.g., bronchoscopy and chest radiography in this case) were required. Although further examination is needed for definite diagnosis, lung POCUS can help to make a possible diagnosis within minutes and helps if immediate treatment is needed.

Therefore, in cases of perioperative hypoxemia, lung POCUS can help to make a quick diagnosis. Checking upper and lower BLUE-points and PLAPS-points is standardized for lung POCUS [6]. Although further evaluation might be needed for a definite diagnosis, lung POCUS is still an efficient, low cost, and irradiative diagnostic tool for anesthesiologists during perioperative hypoxemia. With the improvement of handheld ultrasound systems, the use of ultrasound may be more convenient. In the future, POCUS will continue to play an important role in perioperative and critical care [9].

## 4. Conclusions

In conclusion, we reported the use of lung POCUS to differentiate acute hypoxemia during anesthesia. The bedside ultrasound provides a quick and reliable modality to detect possible causes, including pneumothorax and pneumonia complicated with obstructive atelectasis.

## Data Availability

Not applicable.
